# Rate and Risk Factors Associated With Prolonged Opioid Use After Surgery: A Systematic Review and Meta-analysis

**DOI:** 10.1001/jamanetworkopen.2020.7367

**Published:** 2020-06-25

**Authors:** Oluwadolapo D. Lawal, Justin Gold, Amala Murthy, Rupam Ruchi, Egle Bavry, Anne L. Hume, Adam K. Lewkowitz, Todd Brothers, Xuerong Wen

**Affiliations:** 1 Department of Pharmacy Practice, University of Rhode Island College of Pharmacy, Kingston; 2 Division of Nephrology, Hypertension, and Renal Transplantation, University of Florida, Gainesville; 3 Pain Medicine Section, Anesthesiology Service, Malcom Randall VA Medical Center, Gainesville, Florida; 4 Department of Family Medicine, Warren Alpert Medical School of Brown University, Providence, Rhode Island; 5 Department of Obstetrics and Gynecology, Warren Alpert Medical School of Brown University, Women and Infants Hospital of Rhode Island, Providence; 6 Roger Williams Medical Center, Providence, Rhode Island

## Abstract

**Importance:**

Prolonged opioid use after surgery may be associated with opioid dependency and increased health care use. However, published studies have reported varying estimates of the magnitude of prolonged opioid use and risk factors associated with the transition of patients to long-term opioid use.

**Objectives:**

To evaluate the rate and characteristics of patient-level risk factors associated with increased risk of prolonged use of opioids after surgery.

**Data Sources:**

For this systematic review and meta-analysis, a search of MEDLINE, Embase, and Google Scholar from inception to August 30, 2017, was performed, with an updated search performed on June 30, 2019. Key words may include *opioid analgesics, general surgery, surgical procedures, persistent opioid use,* and *postoperative pain*.

**Study Selection:**

Of 7534 articles reviewed, 33 studies were included. Studies were included if they involved participants 18 years or older, evaluated opioid use 3 or more months after surgery, and reported the rate and adjusted risk factors associated with prolonged opioid use after surgery.

**Data Extraction and Synthesis:**

The Meta-analysis of Observational Studies in Epidemiology (MOOSE) and Preferred Reporting Items for Systematic Reviews and Meta-analyses (PRISMA) reporting guidelines were followed. Two reviewers independently assessed and extracted the relevant data.

**Main Outcomes and Measures:**

The weighted pooled rate and odds ratios (ORs) of risk factors were calculated using the random-effects model.

**Results:**

The 33 studies included 1 922 743 individuals, with 1 854 006 (96.4%) from the US. In studies with available sex and age information, participants were mostly female (1 031 399; 82.7%) and had a mean (SD) age of 59.3 (12.8) years. The pooled rate of prolonged opioid use after surgery was 6.7% (95% CI, 4.5%-9.8%) but decreased to 1.2% (95% CI, 0.4%-3.9%) in restricted analyses involving only opioid-naive participants at baseline. The risk factors with the strongest associations with prolonged opioid use included preoperative use of opioids (OR, 5.32; 95% CI, 2.94-9.64) or illicit cocaine (OR, 4.34; 95% CI, 1.50-12.58) and a preoperative diagnosis of back pain (OR, 2.05; 95% CI, 1.63-2.58). No significant differences were observed with various study-level factors, including a comparison of major vs minor surgical procedures (pooled rate: 7.0%; 95% CI, 4.9%-9.9% vs 11.1%; 95% CI, 6.0%-19.4%; *P* = .20). Across all of our analyses, there was substantial variability because of heterogeneity instead of sampling error.

**Conclusions and Relevance:**

The findings suggest that prolonged opioid use after surgery may be a substantial burden to public health. It appears that strategies, such as proactively screening for at-risk individuals, should be prioritized.

## Key Points

**Question:**

What are the rate and risk factors associated with prolonged use of opioid medications after surgery?

**Findings:**

In this systematic review and meta-analysis of 33 observational studies including more than 1.9 million patients, 7% of patients continued to fill opioid prescriptions more than 3 months after surgery. Preoperative use of opioids, illicit cocaine use, and pain conditions before surgery had the strongest associations with prolonged opioid use after surgery.

**Meaning:**

The findings suggest that an evaluation of opioid use among patients before surgery and modification of patient-level risk factors when possible may be included as part of a comprehensive strategy to reduce the risk of prolonged opioid use after surgery.

## Teaser

This systematic review and meta-analysis assess the rate and patient-level factors associated with increased risks of prolonged use of opioid medications after surgery.

## Introduction

The misuse, overdose, and abuse of prescription opioids constitute sources of substantial morbidity and mortality in the US and globally.^1,2,3,4,5^ Approximately 130 individuals in the US die each day of opioid overdose, with one of the largest proportion of preventable deaths in the US being attributable to opioid-related deaths.^3^ In addition to the substantial mortality burden, prescription opioid misuse, abuse, dependence, and overdose were reported to cost the US health care system an estimated $78.5 billion in 2013.^6^ Increases in prescription opioid use and incidence of opioid-related deaths have also been reported globally, including in European countries and Canada.^4,5,7,8,9^

Many of the efforts to curb the opioid crisis in the US have focused on regulatory changes regarding opioid use for chronic, noncancer pain, with the guidance for postoperative opioid analgesia use being less clear.^10,11,12^ The medical literature^12,13^ purports that inappropriate opioid prescribing for peri- and postoperative analgesia in the form of inadequate or excessive dispensing may contribute to the ongoing epidemic. Of note, opioids remain the standard of care for treatment of acute and routine postoperative pain,^14,15^ and surgical procedures remain the primary reason for exposure to these medications.^12,16^ There is also substantial variation in opioid prescribing among clinicians, particularly in the quantities and dosages of opioids after common general surgical procedures.^17,18^ This variation is further complicated with the potential for misuse and diversion, with 67% to 92% of all opioids prescribed for postoperative pain remaining unused.^19^

The association between opioid prescribing after surgery and the opioid crisis is complex. Inadequate postoperative pain management, including using opioids, has been reported to be associated with increased risk for chronic pain, thus warranting the need for long-term opioid use.^20,21,22,23,24,25^ Conversely, the receipt of prescription opioids after surgery is suggested to be associated with increased risk for chronic opioid use. In a retrospective analysis of population-based claims data from Canada,^26^ individuals prescribed opioids within 7 days of a low-risk surgical procedure were 44% more likely to become prolonged opioid users within 1 year after surgery compared with individuals who did not receive these medications. Lastly, undergoing a surgical procedure has been hypothesized as an independent risk factor for prolonged opioid use after surgery.^11,12,27,28,29^

Although several studies have sought to quantify the rate of and characterize risk factors for prolonged opioid use after surgery, the extent and strength of association have been inconsistent. Despite using similar definitions of prolonged opioid use and eligibility criteria, studies^30,31,32^ enrolling opioid-naive patients undergoing major surgical procedures in the US reported incidence rates ranging from 0.5% to 13.0%. Incidence rates as high as 44% for 1 year after surgery have also been reported.^33,34^ To address these conflicting results and to account for potential bias related to differences in study-level factors and low sample sizes, we performed a meta-analysis of published literature to systematically characterize and aggregate the magnitude and patient-level risk factors associated with increased risk of prolonged opioid use after surgery.

## Methods

This systematic review and meta-analysis was conducted according to the Meta-analysis of Observational Studies in Epidemiology (MOOSE) and Preferred Reporting Items for Systematic Reviews and Meta-analyses (PRISMA) reporting guidelines.^35,36^ The study protocol is available in the PROSPERO database (CRD42019129239).

### Literature Search

Relevant studies were identified through an initial literature search of MEDLINE, Embase, and Google Scholar from inception of these databases to August 30, 2017, with an updated search performed on June 30, 2019. Eligible studies were identified from electronic databases using search terms and keywords such as *opioid analgesics, general surgery, surgical procedures, persistent opioid use,* and *postoperative pain*. The full search strategy is available in the eAppendix in the Supplement. We also searched bibliographies of relevant articles to identify additional eligible publications.

### Study Selection

Two of us (J.G. and A.M.) independently assessed all titles and abstracts of studies to determine studies eligible for full-text review. Eligible studies were restricted to published observational studies evaluating opioid use after surgery. Studies were included if they (1) were published in the English language; (2) enrolled participants 18 years or older; (3) included a minimum of 50 patients; (4) involved a noninjectable opioid prescription fill at least 3 months after the index surgical procedure; and (5) reported the rate and adjusted outcome estimates for patient-level risk factors associated with prolonged opioid use after surgery. Given differences with opioid use in cancer vs noncancer pain management, we excluded studies evaluating cancer pain. In addition, eligible studies needed to have accounted for opioids dispensed in the perioperative period or to have incorporated a lag period for at least 1 month after the index surgical procedure. This criterion was included to account for opioids prescribed as part of routine management of postoperative pain before assessing prolonged opioid use after surgery.

Currently, there is not an accepted definition of prolonged opioid use in the medical literature. Therefore, all studies that met the inclusion criteria were considered regardless of variations in the operational definition of prolonged opioid use within and among studies. However, because opioids are often prescribed preemptively to manage peri- or postoperative pain during the few days or months, in some instances, after surgery, we set a 3-month threshold after the index surgical procedure before assessing prolonged opioid use. As such, prolonged opioid use in this study refers to any opioid use pattern reported by the included studies occurring at least 3 months after surgery. An exception is use of the term *chronic opioid use*. Based on previous literature,^2,11,37,38,39,40^ we defined *chronic opioid use* as the receipt of at least 10 opioid prescription fills, at least 90 consecutive days’ supply of opioids, or 120 cumulative days in the first year after surgery, excluding the initial 90 postoperative days. Because we expected substantial between-study variation in prolonged opioid use definitions, in sensitivity analyses, we repeated our primary analysis to assess the pooled rate of prolonged opioid use by aggregating evidence across studies involving comparable definitions for opioid use after surgery (eTable 1 in the Supplement).

### Data Extraction

Data extraction was performed by the same 2 reviewers (J.G. and A.M.) from the literature search using structured forms. A third reviewer (O.D.L.) assessed the data extraction forms for completeness and accuracy. Extracted information from eligible studies included the study design, sample size, length of follow-up, types of surgical procedure, proportions of opioid-naive and opioid-experienced individuals at baseline, and the definitions of prolonged opioid use after surgery. In addition, rates and adjusted estimates associated with the longest follow-up time were extracted. We did not contact authors for information missing from published texts.

### Quality Assessment

The quality of included studies was assessed by 2 independent reviewers (O.D.L. and J.G.) using the Newcastle-Ottawa Scale,^41^ and disagreements were resolved by discussion. Studies with a Newcastle-Ottawa Scale score greater than 7 were considered to be high in quality.

### Statistical Analysis

The primary outcomes of interest were the pooled rate and magnitude for individual risk factors of prolonged opioid use after surgery. No restrictions were made in the type of effect estimates extracted; therefore, studies reporting odds ratios (ORs), risk ratios, or hazard ratios were eligible for inclusion. Based on the overall low prevalence of the risk factors in the individual studies (ie, <10%), we regarded risk ratios and ORs as equivalent risk measures. However, we pooled studies reporting hazard ratios in a separate analysis. We calculated the pooled rate of prolonged opioid use after surgery weighted by the sample size of each eligible study. When 2 or more studies reported adjusted estimates for the same risk factor, a pooled OR and the corresponding 95% CI were estimated using the inverse variance method with a random-effects model.^42^ Based on an a priori assumption of substantial between-study variation, we prespecified to use the random-effects model for all meta-analyses. Between-study heterogeneity was tested using the Cochran Q statistic^43^ and quantified by the *I*^2^ value.^44^ We considered heterogeneity to be significant at *P* < .10 and *I*^2^>50% to indicate substantial between-study variation that was beyond chance.^45^ Heterogeneity was further assessed in sensitivity analyses. Small-study effect, commonly referred to as publication bias, was examined using a funnel plot and the Egger regression test. Except for heterogeneity, statistical significance was set at *P* < .05; all tests were 2-sided. Statistical analyses were conducted using Comprehensive Meta-Analysis Software, version 3.0 (Biostat).

We performed additional analyses to evaluate the potential sources of heterogeneity and robustness of the primary findings. First, we restricted our analyses to studies enrolling only opioid-naive patients before surgery. We accepted definitions of opioid naive from each eligible study. The definitions of opioid naivety in studies enrolling only opioid-naive participants are presented in eTable 2 in the Supplement. Second, we examined whether the rate of prolonged opioid use after surgery differed by source population or insurance plan, follow-up period (≤6 months vs >6 months), type of surgery (major vs minor surgery and orthopedic vs nonorthopedic surgery), and studies conducted in the US vs non-US countries to assess whether our main finding was moderated by potential differences in prescribing patterns across countries. Classification of major or minor surgery was based on previously published reports^11,12,46^ and expert opinion (E.B.) (eTable 3 in the Supplement). We then aggregated evidence across studies involving comparable definitions for chronic or prolonged opioid use after surgery. Lastly, for each risk factor reported by at least 3 studies, we recalculated the pooled effect by omitting 1 study at a time. This leave-one-out analysis was performed to determine the influence of an individual study on the pooled effects.

## Results

### Study Selection

**Figure 1.**
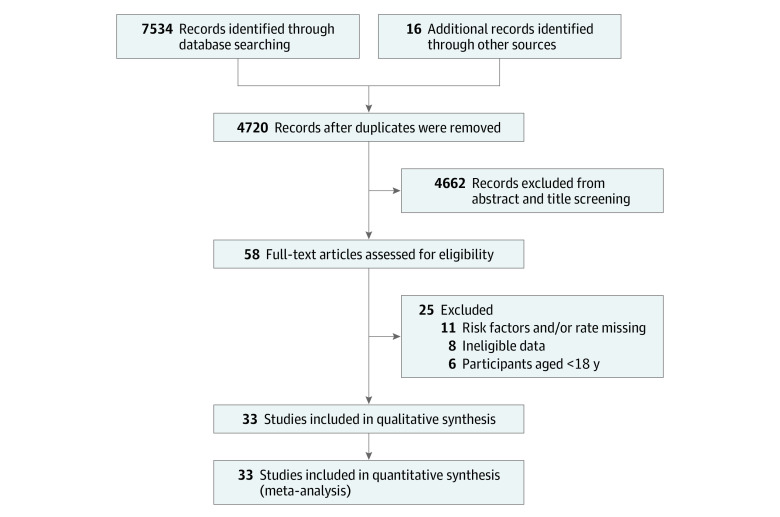
PRISMA Flow Diagram of Included Studies

The search of electronic databases yielded 7534 citations. After removal of duplicates and full-text reviews, a total of 33 studies involving 1 922 743 individuals, with 1 854 006 (96.4%) from the US.^11,12,16,27,29,30,31,32,33,46,47,48,49,50,51,52,53,54,55,56,57,58,59,60,61,62,63,64,65,66,67,68,69^ The PRISMA diagram is shown in Figure 1.

### Study Characteristics

Characteristics of the eligible studies are presented in Table 1. Sample sizes of included studies ranged from 109 to 675 527 participants. The included studies were conducted in Australia, Canada, Denmark, France, the US, and the UK, with 26 (78.8%) from the US. Of the 1 247 216 individuals enrolled in 32 studies with sex information,^11,12,16,27,29-33,46,48-69^ 1 031 399 (82.7%) were females. Information on age was available in 22 studies^11,12,27,29,30,33,48,50,53,54,58-60,62-69^; the mean (SD) age of participants was 59.3 (12.8) years. The minimum age of participants was 39.0 years and the maximum age was 80.0 years. Of the 33 studies, 14 (42.4%) involved data from commercial insurance plans, 12 (36.4%) used hospital institution data, 5 (15.2%) involved military or veterans’ insurance plans, and the remaining 2 (6.1%) were based on data from national, publicly funded health care systems. Eight studies (24.2%) enrolled only opioid-naive participants,^11,12,16,30,31,32,46,50^ 18 (54.5%) enrolled both opioid-naive and opioid-experienced participants,^27,29,33,48,49,51,52,55,56,57,58,59,60,61,66,70^ and the remaining 7 studies lacked sufficient information to categorize participants.^47,53,54,62,63,64,65^ The definitions of *long-term*, *persistent*, and *prolonged opioid use* and *opioid naivety* varied across studies (Table 1, and eTables 1-2 in the Supplement). Subgroup analysis based on quality was not performed because the individual summed score from the Newcastle-Ottawa-Scale varied between 7 and 9, suggesting that all included studies were high quality.

**Table 1.** Characteristics of Included Studies

Source	Country	Type of surgery	Data source or study setting	Participants, No.	Median age at baseline, y	Enrollment period	No. (%)			Type of prolonged opioid use and definition	Follow-up time	NOWS score
							Opioid-naive participants before surgery	Participants with prolonged opioid use	Opioid-naive participants			
Bateman et al,^16^ 2016	US	Cesarean delivery	Commercial insurance beneficiaries	80 127	NR	January 1, 2003, to December 31, 2011	80 127 (100)	285 (0.4)	285 (0.4)	Persistent use; using a trajectory model with 5 patient groups based on probability of an opioid fill during each of 12 consecutive 30-d follow-up; opioid fill in ≥4, 6, or 8 mo of follow-up	1 y	9
Raebal et al,^27^ 2014	US	Bariatric surgery	Commercial insurance beneficiaries	10 643	47	January 1, 2005, to December 31, 2009	6483 (60.9)	421 (4.0)	84 (1.3)	Chronic opioid use defined as ≥10 fills dispensing ≥90 consecutive days or ≥120-d total supply	1 y	9
Clarke et al,^46^ 2014	Canada	Major elective surgeries	National health care system databases	39 140	NR	April 1, 2003, to March 31, 2010	39 140 (100)	1229 (3.1)	1229 (3.1)	Prolonged opioid use defined as the filling of opioid fills within the first 90 d after surgery and ≥1 opioid fill for 91-180 d after surgery	6 mo	9
Shah et al,^47^ 2017	US	Urologic surgery	Hospital institution data	675 527	62	2007-2011	NR	608 (0.1)	NR	Opioid dependence; outcome was assessed on the presence of *ICD-9* diagnosis codes	1 y	9
Sun et al,^11^ 2016	US	Total knee arthroplasty, total hip arthroplasty, laparoscopic cholecystectomy, open cholecystectomy, laparoscopic appendectomy, open appendectomy, cesarean delivery, functional endoscopic sinus surgery, cataract surgery, transurethral prostate resection, and simple mastectomy	Commercial insurance beneficiaries	641 941	44	Jan 1, 2001, to Dec 31, 2013	641 941 (100)	2039 (0.3)	2039 (0.3)	Chronic opioid use defined as ≥10 opioid fills or ≥120-d supply of an opioid in the first 365 d after surgery, excluding the first 90 d after surgery	1 y	9
Connolly et al,^48^ 2017	US	Lumbar spinal fusion surgery	Commercial insurance beneficiaries	8377	49.6	January 1, 2009, to December 31, 2012	1332 (15.9)	2458 (29.3)	29 (2.2)	Long-term opioid use defined as opioid fill at least 365 d in 24 mo after surgery	2 y	9
Bedard et al,^49^ 2017	US	Total knee arthroplasty	Commercial insurance beneficiaries	73 959	NR	January 1, 2007, to December 31, 2014	29 801 (40.3)	8780 (11.8)	969 (3.2)	Prolonged opioid use defined as opioid fill each mo for up to 1 y after surgery	1 y	9
Johnson et al,^31^ 2016	US	Hand surgery	Commercial insurance beneficiaries	59 725	NR	January 1, 2010, to December 31, 2012	59 725 (100)	7764 (13.0)	7764 (13.0)	Prolonged opioid use defined as ≥1 opioid fill between 30 d before and 2 wk after surgery and ≥1 opioid prescription 90-180 d after surgery	6 mo	9
Schoenfeld et al,^50^ 2017	US	Spine surgery: discectomy, decompression, lumbar posterolateral arthrodesis, or lumbar interbody arthrodesis	Military insurance beneficiaries, TRICARE	9991	46.4	January 1, 2006, to December 31, 2014	9991 (100)	2 (0.02)	2 (0.02)	Sustained opioid use defined as continued opioid use up to 6 mo after surgery	1 y	9
Westermann et al,^51^ 2017	US	Rotator cuff repair	Commercial insurance beneficiaries	35 155	NR	January 1, 2007, to December 31, 2014	19 925 (56.7)	6749 (19.2)	1594 (8.0)	Prolonged opioid use defined as opioid fill each mo up to 1 y after surgery	3 mo	9
Rosenbloom et al,^53^ 2017	Canada	Traumatic musculoskeletal injury and corrective surgery	Hospital institution data	122	44.8	May 2012 to July 2013	NR	43 (35.2)	NR	Persistent opioid use defined as ≥1 opioid fill 4 mo after surgery	4 mo	8
Brummett et al,^12^ 2017	US	Surgical procedure: major, ventral incisional hernia repair, colectomy, reflux surgery, bariatric surgery, and hysterectomy; minor, varicose vein removal, laparoscopic cholecystectomy, laparoscopic appendectomy, hemorrhoidectomy, thyroidectomy, transurethral prostate surgery, parathyroidectomy, and carpal tunnel	Commercial insurance beneficiaries	36 177	44.6	January 1, 2012, to June 30, 2015	36 177 (100)	2176 (6.0)	2176 (6.0)	Persistent opioid use defined as opioid fill 90-180 d after surgery	6 mo	9
Fuzier et al,^54^ 2018	France	Carpal tunnel surgery	National health care system	3665	58	January 1 to June 30, 2010	NR	183 (5.0)	NR	Prolonged opioid use defined as opioid fill each month from 2 mo before surgery to >2 to 12 mo after surgery	1 y	9
Carroll et al,^55^ 2012	US	Mastectomy, lumpectomy, thoracotomy, and total hip or knee replacement	Hospital institution data	109	NR	January 2007 to April 2009	21 (19.3)	6 (5.5)	NR	Opioid use 150 d after surgery	150 d	8
Pugely et al,^56^ 2018	US	Cervical spine surgery	Commercial insurance beneficiaries	17 391	NR	2007-2015	8278 (47.6)	4128 (45.3)	522 (6.3)	Prolonged opioid use defined as opioid prescription fill per month for 1 y after surgery	1 y	9
Politzer et al,^57^ 2018	US	Total knee arthroplasty	Commercial insurance beneficiaries	66 950	NR	2007-2013	30 282 (45.2)	12 760 (34.6)	1514 (5.0)	Chronic opioid use defined as opioid fill for 6 contiguous months after surgery	2 y	9
Swenson et al,^32^ 2018	US	Hysterectomy	Commercial insurance beneficiaries	24 331	NR	January 1, 2011, to December 31, 2014	24 331 (100)	122 (0.5)	122 (0.5)	Persistent opioid use defined as ≥2 opioid fills within 6 mo after surgery with ≥1 fill every mo and either total oral morphine equivalent of at least 1500 or at least 39 d of opioid supply	6 mo	8
Namba et al,^33^ 2018	US	Total knee arthroplasty	Hospital institution data	23 726	68	January 1, 2008, to December 31, 2011	14 236 (40.0)	7218 (30.4)	NR	Prolonged opioid use defined as number of opioid fills every 90-d period 1 y after surgery	1 y	9
Bennett et al,^30^ 2019	US	Body contouring surgical procedures after bariatric surgery	Commercial insurance beneficiaries	11 257	41.0	January 1, 2002, to September 30, 2014	11 257 (100)	690 (6.1)	690 (6.1)	Persistent opioid use defined as continued opioid fills 90-180 d after surgery among patients who had opioid fills perioperatively	6 mo	8
Westermann et al,^52^ 2019	US	Hip arthroscopy	Commercial insurance beneficiaries	1208	NR	January 1, 2007, to June 30, 2016	698 (57.8)	183 (15.1)	37 (5.3)	Prolonged opioid use defined as opioid fills each mo up to 1 y after surgery	1 y	9
Goesling et al,^58^ 2016	US	Total knee and total hip arthroplasty	Hospital institution data	574	63.3	March 2010 to May 2013	407 (70.9)	70 (12.2)	20 (4.9)	Persistent opioid use defined as opioid fills at 6 mo after surgery	6 mo	7
Lindestrand et al,^59^ 2015	Denmark	Hip fracture surgery	Hospital institution data	413	79.5	May 30, 2010, to March 31, 2011	314 (76.0)	124 (30.2)	9 (2.9)	Persistent opioid use defined as opioid fills 90 and 180 d after surgery	6 mo	9
Mulligan et al,^60^ 2016	US	Ankle and foot reconstruction	Hospital institution data	132	55	NR	89 (67.4)	52 (39.4)	14 (15.7)	Defined as continued opioid use for 3 mo after surgery	1 y	8
Rao et al,^61^ 2018	US	Shoulder arthroplasty	Hospital institution data	4243	NR	January 1, 2008, to December 31, 2014	1061 (25.0)	1598 (40.0)	NR	Persistent opioid use defined as opioid use after surgery within 1 y of surgery, evaluated quarterly	1 y	9
Singh and Lewallen,^62^ 2010	US	Primary total hip arthroplasty	Hospital institution data	3005	64.9	1993-2005	NR	85 (2.8)	NR	Opioid use after surgery assessed by validated questionnaire given 2 and 5 y after index surgery	2 and 5 y	7
Singh and Lewallen,^63^ 2012	US	Primary total knee arthroplasty	Hospital institution data	4234	68	1993-2005	NR	61 (1.4)	NR	Opioid use after surgery assessed by validated questionnaire given 2 and 5 y after index surgery	2 and 5 y	7
Singh and Lewallen,^64^ 2014	US	Revision total knee arthroplasty	Hospital institution data	881	69	1993-2005	NR	52 (5.9)		Opioid use after surgery assessed by validated questionnaire given 2 and 5 y after index surgery	2 and 5 y	7
Valdes et al,^65^ 2015	UK	Total joint replacement	Hospital institution data	852	73.7	2008-2014	NR	215 (25.1)	NR	Opioid use after surgery assessed by questionnaire given 1.27 y after index surgery	4 y	7
Rozet et al,^66^ 2014	US	Knee arthroscopy	Military insurance beneficiaries, Veterans Affairs	145	39	2007-2010	82 (56.6)	43 (30.0)	NR	Prolonged opioid use defined as opioid fills uninterruptedly for >3 mo after surgery	3.5 mo	8
Kim et al,^29^ 2017	US	Hip or knee arthroplasty	Commercial insurance beneficiaries	57 545	61.5	January 1, 2004, to December 31, 2013	7425 (12.9)	4373 (7.6)	48 (0.6)	Persistent opioid use defined as ≥1 opioid fill each mo during the 1 y after surgery based on group-based trajectory models	1 y	9
Hansen et al,^67^ 2017	Australia	Total knee arthroplasty	Military insurance beneficiaries, Australian Government Department of Veterans Affairs	15 020	79	January 1, 2001, to December 31, 2012	9223 (61.4)	787 (5.2)	64 (0.7)	Chronic opioid use defined as >90 d of continuous opioid use or >120 d of cumulative use	1 y	9
Inacio et al,^68^ 2016	Australia	Total hip arthroplasty	Military insurance beneficiaries, Australian Government Department of Veterans Affairs	9525	80	January 1, 2001, to December 31, 2012	5138 (53.9)	492 (5.2)	38 (0.7)	Chronic opioid use defined as >90 d of continuous opioid use or >120-d cumulative use	1 y	9
Hadlandsmyth et al,^69^ 2018	US	Total knee arthroplasty	Military insurance beneficiaries, Veterans Affairs	6653	66	2013-2015	5322 (80.0)	866 (13.0)	107 (2.0)	Chronic opioid use defined as continuous opioid fills assessed 3, 6, and 12 mo after surgery	1 y	8

Abbreviations: *ICD*, *International Classification of Diseases*; NOW, Newcastle-Ottawa Scale; NR, not reported.

### Primary Outcomes

**Figure 2.**
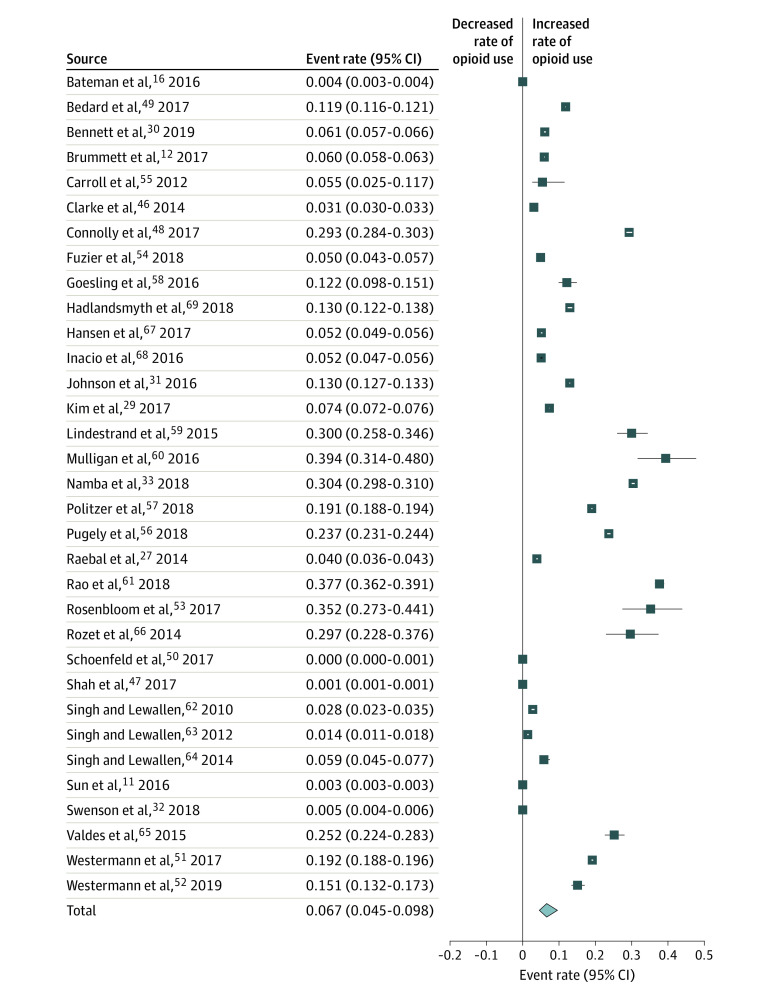
Forest Plot of Studies Assessing Prolonged Opioid Use After Surgery Squares indicate event rates, with horizontal lines representing 95% CIs. The diamond represents the pooled total, with the points of the diamond representing 95% CIs. The data show substantial between-study heterogeneity (*P* < .001; *I^2^* = 99.96%).

Across the 33 eligible studies based on random-effects analysis, the pooled rate of prolonged opioid use after surgery was 6.7% (95% CI, 4.5%-9.8%) (Figure 2), with substantial between-study heterogeneity (*P* < .001; *I^2^* = 99.96%).

With the exception of anxiety, we were unable to find comparable risk factors across the 2 studies^50,55^ that used hazard ratios. Therefore, our analyses on risk factors for prolonged opioid use were derived from studies reporting risk ratios or ORs.

Significantly increased risks were observed among females compared with males (OR, 1.16; 95% CI, 1.08-1.25) and among individuals with a high school degree vs a college degree or higher (OR, 1.20; 95% CI, 1.04-1.37) (Table 2).

**Table 2.** Baseline Characteristics Associated With Prolonged Opioid Use After Surgery

Characteristic	Studies, No.	References	Random-effects pooled OR (95% CI)	*I*^2^, %	*P* value
**Demographic characteristic**					
Age					
≥50 y	NA	NA	1 [Reference]	NA	NA
<50 y	2	49, 56	1.83 (0.98-3.48)	98.71	<.001
Body mass index^a^					
<25	NA	NA	1 [Reference]	NA	NA
25-29.9	4	62-64, 69	1.04 (0.52-2.10)	41.63	.16
30-34.9	3	62-64	1.21 (0.61-2.38)	38.58	.20
35-39.9	3	62-64	0.63 (0.31-1.30)	0	.54
≥40	3	62-64	0.98 (0.42-2.33)	17.52	.26
Sex					
Male	NA	NA	1 [Reference]	NA	NA
Female	14	12, 31, 33, 46, 48, 49, 54, 57, 58, 61, 62, 65, 67, 69	1.16 (1.08-1.25)	88.87	<.001
Race/ethnicity					
White	NA	NA	1 [Reference]	NA	NA
African American	7	12, 27, 32, 33, 47, 61, 69	1.02 (0.92-1.13)	69.35	.01
Asian	4	12, 33, 47, 61	0.68 (0.45-1.03)	91.25	<.001
Hispanic	5	12, 27, 33, 47, 61	0.92 (0.80-1.05)	79.85	<.001
Educational level					
College degree or more	NA	NA	1 [Reference]	NA	NA
Less than high school	2	12, 30	1.06 (0.56-2.00)	0	.91
High school	2	12, 30	1.20 (1.04-1.37)	0	.63
Some college	2	12, 30	1.10 (0.92-1.33)	49.41	.16
Preoperative medication use					
Antidepressants	6	11, 16, 27, 29, 46, 69	1.42 (1.11-1.81)	90.97	<.001
Antipsychotics	2	11, 16	1.15 (0.90-1.48)	0	.74
Benzodiazepines	5	11, 16, 29, 46, 69	1.53 (1.20-1.95)	91.72	<.001
Opioids	14	12, 27, 29, 32, 33, 51, 52, 56, 58-61, 66, 69	5.32 (2.94-9.64)	99.57	<.001
Substance use					
Alcohol	8	11, 12, 29, 31, 47, 49, 53, 60	1.55 (1.07-2.25)	90.74	<.001
Cocaine	2	16, 29	4.34 (1.50-12.58)	0	.64
Marijuana	2	16, 29	0.89 (0.29-2.74)	0	.36
Tobacco	10	12, 16, 27, 29-31, 47-49, 60	1.55 (1.23-1.96)	95.16	<.001
**Medical comorbidities**					
Mental health conditions					
Anxiety	9	12, 27, 30, 33, 53, 61-64	1.14 (1.06-1.23)	61.10	<.001
Bipolar disorders	2	33, 61	0.88 (0.79-0.98)	0	>.99
Depression	15	11, 32, 33, 47-49, 52, 53, 56, 61-64, 68, 71	1.54 (1.25-1.91)	97.90	<.001
Mood disorders	3	12, 30, 60	1.85 (1.11-3.07)	92.44	<.001
Psychiatric disorders	5	11, 30, 51, 61, 68	1.04 (0.95-1.13)	0	.75
Posttraumatic stress disorder	2	33, 61	1.39 (1.21-1.59)	0	.37
Unspecified mental disorders	5	12, 30, 31, 33, 54	1.45 (0.78-2.68)	53.40	.07
Pain conditions					
Arthritis	4	12, 30, 33, 61	1.19 (0.93-1.52)	94	<.001
Back pain	11	12, 16, 29, 33, 49, 51, 56, 61, 65, 67, 68	2.05 (1.63-2.58)	98.65	<.001
Chronic pain	5	27, 53, 60, 61, 69	1.35 (1.04-1.75)	85.92	<.001
Fibromyalgia	7	16, 29, 33, 49, 51, 56, 61	1.43 (1.15-1.79)	97.41	<.001
Migraine	4	16, 29, 61, 68	1.36 (1.02-1.80)	80.87	.01
Neck pain	4	12, 30, 33, 61	1.12 (1.02-1.23)	60.17	.06
Osteoarthritis	3	33, 54, 61	1.03 (0.96-1.09)	0	.47
Unspecified pain disorders	9	12, 30-33, 54, 58, 61, 65	1.45 (1.21-1.72)	63.24	<.001
Other medical conditions					
Anemia	2	33, 61	1.06 (1.01-1.12)	0	.88
Coagulopathy	2	33, 61	1.20 (1.08-1.34)	0	.74
Cerebrovascular disease	2	46, 47	0.90 (0.55-1.46)	34.07	.22
Diabetes	5	46, 54, 60, 61, 68	1.09 (0.90-1.31)	59.42	.04
Hypothyroidism	2	33, 61	0.99 (0.95-1.04)	0	.87
Liver disease	4	33, 47, 61, 68	1.23 (0.98-1.54)	87.56	.005
Obesity	2	48, 65	1.25 (0.67-2.35)	87.56	.005
Pulmonary disease	4	48, 65	1.32 (1.07-1.63)	92.55	<.001
Renal disease	3	33, 46, 61	1.02 (0.97-1.08)	0	.40
Substance abuse	11	11, 16, 27, 29, 31, 33, 49, 53, 56, 61, 69	1.58 (1.14-2.21)	98.47	<.001

Abbreviations: NA, not applicable; OR, odds ratio.

^a^ Calculated as weight in kilograms divided by height in meters squared.

Increased risk of prolonged opioid use was associated with use of antidepressants, opioids, benzodiazepines, alcohol, cocaine, or tobacco before surgery (Table 2). Preoperative use of opioids (OR, 5.32; 95% CI, 2.94-9.64), tobacco (OR, 1.55; 95% CI, 1.23-1.96), or cocaine (OR, 4.34; 95% CI, 1.50-12.58) were identified as having the strongest associations with prolonged use of opioids after surgery.

Medical comorbidities were differentiated into 3 specific categories: psychological, pain-associated conditions, and a broader category composed of disorders such as diabetes, pulmonary disease, and obesity (Table 2). When evaluating the association between psychological disorders before surgery and prolonged opioid use after surgery, increased risks were observed among participants with diagnoses of anxiety (OR, 1.14; 95% CI, 1.06-1.23), depression (OR, 1.54; 95% CI, 1.25-1.91), and mood disorders (OR, 1.85; 95% CI, 1.11-3.07). In contrast, patients with a diagnosis of bipolar disorder before surgery had significantly lower risks for prolonged opioid use after surgery (OR, 0.88; 95% CI, 0.79-0.98). Among pain conditions aggregated across all studies, prolonged opioid use after surgery was most strongly associated with a history of back pain (OR, 2.05; 95% CI, 1.63-2.58) and fibromyalgia (OR, 1.43; 95% CI, 1.15-1.79) (Table 2).

### Sensitivity and Additional Analyses

Our primary findings remained largely unchanged in leave-one-out analyses (eTable 4 in the Supplement). No evidence of publication bias was found with the Egger regression-based test (intercept, –20.99; 95% CI, –46.04% to 4.07%; SE, 12.28; *P* = .10) (eFigure in the Supplement). Studies involving only opioid-naive participants before surgery had lower pooled rates of prolonged opioid use after surgery (1.2%; 95% CI, 0.4%-3.9%). In the restricted analysis assessing chronic opioid use,^11,50,57,67,68,69,71^ we observed a pooled rate of 2.3% (95% CI, 0.5%-10.6%). Similarly, in the 10 studies^12,30,31,33,46,53,55,58,59,61^ defining prolonged opioid use as the filling of at least 1 opioid prescription within the first 90 days after surgery and the filling of at least 1 additional opioid prescription from 91 to 180 days after surgery, the pooled rate was 13.8% (95% CI, 7.9%-23.0%). No significant difference was observed in a comparison of major vs minor surgical procedures (pooled rate, 7.0%; 95% CI, 4.9%-9.9% vs 11.1%; 95% CI, 6.0%-19.4%; (*P* = .20). Results from meta-analyses of other study-level factors are presented in Table 3.

**Table 3.** Random-Effects Pooled Rates From Additional Analyses

Characteristic	Studies, No.	Participants, No.	References	Pooled rate (95% CI)	*P* value	*I*^2^, %	*P* value
Country of patient enrollment							
US	26	1 854 006	12, 16, 27, 29-33, 47-52, 55-58, 60-64, 66, 69	5.8 (3.7-9.0)	.09	99.97	<.001
Non-US	7	68 737	46, 53, 54, 59, 65, 67, 68	10.7 (6.2-18.1)		99.56	<.001
Length of follow-up, mo							
>6	22	1 715 595	11, 16, 27, 29, 33, 47-50, 52, 54, 56, 57, 60-65, 67-69	5.4 (3.1-9.5)	.12	99.97	<.001
≤6	11	207 148	12, 30-32, 46, 51, 53, 55, 58, 59, 66	9.6 (6.2-14.6)		99.86	<.001
Source population or study setting							
Commercial insurance beneficiaries	14	1 124 786	11, 12, 16, 27, 29-32, 48, 49, 51, 52, 56, 57	6.0 (3.5-1.2)	NA	99.97	<.001
Hospital institution data	12	713 818	33, 47, 53, 55, 58, 59, 61-65, 72	9.8 (2.5-31.5)		99.95	<.001
Military insurance beneficiaries	5	41 334	50, 66-69	5.4 (2.9-9.8)		99.36	<.001
National insurance plans	2	42 805	46, 54	3.9 (2.5-6.2)		97.18	<.001
Major vs minor surgery							
Major surgery	27	1 228 350	16, 27, 29, 31-33, 46-51, 53, 55-65, 67-69	7.0 (4.9-9.9)	.20	99.94	<.001
Minor surgery	4	16 275	30, 52, 54, 66	11.1 (6.0-19.4)		98.75	<.001
Orthopedic vs nonorthopedic surgery							
Orthopedic	23	399 248	29, 31, 33, 48-54, 56-60, 62-69	12.1 (9.7-14.9)	.07	99.84	<.001
Nonorthopedic	6	806 128	16, 27, 30, 32, 47, 61	1.7 (0.2-13.7)	NA	99.97	<.001
Other study-level analyses							
Only opioid-naive patients before surgery	8	902 689	11, 12, 30-32, 46, 50	1.2 (0.4-3.9)	NA	99.97	<.001
Similar definitions for chronic opioid use after surgery^a^	7	760 723	11, 27, 50, 57, 67-69	2.3 (0.5-1.6)	NA	99.98	<.001
Similar definitions for prolonged opioid use after surgery^b^	10	175 486	12, 30, 31, 33, 46, 53, 55, 58, 59, 61	13.8 (7.9-23.0)	NA	99.93	<.001

Abbreviation: NA, not applicable.

^a^ Included studies defining opioid use after surgery as the receipt of opioids for 10 opioid fills or more, 90 or more consecutive days’ supply of opioids, or 120 cumulative days in the first year after surgery after excluding the first 90 postoperative days.

^b^ Included studies defining prolonged opioid use as 1 opioid fill or more within 91 to 180 days after surgery.

## Discussion

This systematic review and meta-analysis of observational studies^11,12,16,27,29,30,31,32,33,46,47,48,49,50,51,52,53,54,55,56,57,58,59,60,61,62,63,64,65,66,67,68,69^ extend the results of a previous meta-analysis^73^ reporting prolonged opioid use among approximately 1 in 10 individuals undergoing a major or minor surgical procedure. Our analyses indicated that approximately 7% of patients filled opioid prescriptions at 3 months and more than 1 year after surgery, a time beyond the normal postoperative recovery period.^74^ A higher rate was observed when prolonged opioid use was defined as the filling of at least 1 prescription for opioids within 91 to 180 days after surgery. However, our primary pooled rate was attenuated when we restricted our analyses to patients considered as opioid naive before surgery or to studies involving a more conservative definition of prolonged use that is commonly used in the medical literature to characterize chronic opioid use. Although these rates may appear to be relatively low, the negative consequences that prolonged opioid use may impose on public health is perhaps better elucidated when indexed to the number of surgical procedures performed annually in the US. In 2010, approximately 51.4 million inpatient and 48.3 million ambulatory surgical procedures were estimated to have been performed in the US.^75,76^ Based on previous studies^77^ reporting that 4 of 5 patients undergoing surgery receive opioids, our pooled rate of 6.9%, when extrapolated to the total number of surgical procedures, implies that up to 5.7 million Americans may potentially become persistent opioid users annually after surgery. Of note, individuals with prolonged opioid use after surgery constitute a group with potentially significant risk of chronic use. Therefore, prioritizing strategies that mitigate the transition of patients undergoing surgery to persistent opioid use while still optimizing the management of postoperative pain is of importance.

A possible approach to reducing the burden of prolonged opioid use is to characterize the underlying mechanisms, including patient-level risk factors, that may be associated with prolonged and/or chronic use of opioids after surgery. This approach, in part, rests on the assumption that patient-level risk factors associated with prolonged opioid use may be modifiable and can be used in screening for at-risk individuals.^74^ Our results indicate that preoperative exposure to medications, such as opioids, antidepressants, benzodiazepines, or cocaine; demographic factors, such as sex; and presence of medical comorbidities, including chronic pain, back pain, substance abuse, mood disorders, or depression before surgery, had some of the strongest associations with prolonged opioid use after surgery. Congruent with previous reviews,^73,78^ the strongest association in the current study was observed with preoperative opioid use, wherein individuals who had filled at least 1 opioid prescription in the year before surgery had a 5.3-fold risk of prolonged opioid use after surgery (pooled OR, 5.32; 95% CI, 2.94-9.64). These findings of increased risk of preoperative opioid use and prolonged use after surgery was further corroborated when we restricted our analyses to studies enrolling opioid-naive participants at baseline; the pooled rate of prolonged opioid use after surgery decreased more than 5-fold. Appropriate prescribing of the dose and quantity of opioids after surgery, the evaluation of opioid use in patients before surgery, and attempts to modify patient-level risk factors when possible or to treat underlying medical conditions before surgery may be included as part of a comprehensive strategy to reduce prolonged opioid use after surgery. Multimodal analgesia, psychobehavioral management of pain, and regional and neuraxial anesthesia have also been listed in the literature^78,79,80^ as strategies associated with reducing the transition to prolonged opioid use after surgery.

Although our analyses suggest that surgery may be associated with long-term opioid use, it is possible that the observed association was enhanced by confounding from an underlying chronic pain condition, the developing of persistent postsurgical pain, or surgical procedures exacerbating preexisting conditions and thus warranting long-term opioid management. Persistent postsurgical pain is a recognized complication of surgery and has been reported after common surgical procedures, including cesarean delivery or hip replacement.^38,81^ Several studies^38,81,82,83,84^ suggest that between 20% and 60% of individuals who undergo surgical procedures may transition from acute to persistent or chronic postsurgical pain. Because opioids were considered the standard of care for chronic noncancer pain management for studies included in this meta-analysis,^2,10^ the findings suggest that a high rate of prolonged opioid use after surgery may reflect the expected opioid use patterns among individuals with persistent postsurgical pain or underlying chronic pain. Because of a lack of information in the included studies, we were unable to assess the association between these confounding factors and opioid use after surgery in our analyses.

Of note, other mechanisms not associated with surgical pain before or after undergoing the procedure could have explained the findings of increased prolonged opioid use with surgery. Because major surgical procedures are likely to be associated with higher frequencies or intensities of postoperative pain and perhaps with a longer recovery time compared with minor surgical procedures, we expected significant differences in the pooled rate of prolonged opioid use in major vs minor surgical procedures. However, we found no such evidence in our subgroup analysis. Although a similar finding was recently reported in a large retrospective study of US adults undergoing minor or major surgical procedures,^12^ a meta-analysis by Mohamadi et al^72^ reported significant differences in prolonged opioid use between these categories of procedures. Therefore, further research should aim to delineate the causal mechanisms of continuous use of opioids in the postoperative period, particularly in the context of surgical pain.

### Limitations

This study has limitations. Because the studies included in our analyses were observational by design, our findings may be prone to several forms of systematic bias, including selection bias and measurement errors. Of importance, our findings may have been subject to confounding by the underlying indication and inadequate bias adjustment. Second, although we performed several sensitivity analyses to explore the sources of heterogeneity, we were unable to explain the substantial heterogeneity present in most of our analyses. We used a random-effects model for our analyses, with the a priori assumption that the included studies would be heterogenous in their design, sample size, definitions of prolonged opioid use and risk factors, and adjustment of covariates. Third, because of a paucity of eligible studies and suboptimal reporting, we were unable to exclude studies involving participants with chronic opioid use at baseline, participants with preexisting pain disorders, or participants with a diagnosis of cancer before surgery—conditions that are frequently managed with opioids. Of note, the inclusion of these individuals may have led to an overestimation in the magnitude of prolonged opioid use after surgery.^85^ In addition, although less likely to be substantial, it is unknown the extent to which some of the eligibility criteria (eg, requiring studies to have reported the rate and risk factors for prolonged use) or not contacting authors may have affected the magnitude of observed association.

Despite these limitations, confidence in our findings is perhaps reinforced because of the absence of small-study bias and consistent results from study-level factors that might have moderated our observed association. Nevertheless, further research is needed to quantify the effect of these various sources of bias on our study findings.

## Conclusions

In this study, preoperative use of opioids and cocaine and the presence of comorbid pain conditions before surgery had the strongest associations with prolonged opioid use after surgery. These largely modifiable patient-level risk factors may be included as part of a comprehensive strategy to screen for at-risk individuals requiring transition to nonopioid interventions after surgery while ensuring appropriate short-term opioid use to manage postoperative pain. Research is needed to further investigate the association between surgical pain and prolonged opioid use after surgery.

## Notes

Supplement. **eAppendix.** Literature Search Strategy for MEDLINE **eTable 1.** Included Studies Categorized by Definitions for Assessing Opioid Utilization After Surgery **eTable 2.** Operational Definition of Opioid Naivety in Studies Enrolling Only Opioid-Naive Individuals Before Surgery **eTable 3.** Classification of Surgeries into Minor, Major, or Major and Minor Surgeries **eTable 4.** Risk Factors for Prolonged Opioid Use After Surgery (Pooled Estimates After Leave-1-Out Analyses) **eFigure 1.** Funnel Plot Assessing Publication Bias/Small-Study Effects **eReferences.**
