# Supplementary material for: Rate and Risk Factors Associated With Prolonged Opioid Use After Surgery: A Systematic Review and Meta-analysis
Source: JAMA Netw Open. 2020 Jun 25;3(6):e207367. doi: 10.1001/jamanetworkopen.2020.7367 (PMC7317603; doi:10.1001/jamanetworkopen.2020.7367)
Supplement: Supplement. — eAppendix. Literature Search Strategy for MEDLINE eTable 1. Included Studies Categorized by Definitions for Assessing Opioid Utilization After Surgery eTable 2. Operational Definition of Opioid Naivety in Studies Enrolling Only Opioid-Naive Individuals Before Surgery eTable 3. Classification of Surgeries into Minor, Major, or Major and Minor Surgeries eTable 4. Risk Factors for Prolonged Opioid Use After Surgery (Pooled Estimates After Leave-1-Out Analyses) eFigure 1. Funnel Plot Assessing Publication Bias/Small-Study Effects eReferences. [file jamanetwopen-3-e207367-s001.pdf]

# Supplementary Online Content

Lawal OD, Gold J, Murthy A, et al. Rate and risk factors associated with prolonged opioid use after surgery: a systematic review and meta-analysis. *JAMA Netw Open*. 2020;3(6):e207367. doi:10.1001/jamanetworkopen.2020.7367

**eAppendix.** Literature Search Strategy for MEDLINE

**eTable 1.** Included Studies Categorized by Definitions for Assessing Opioid Utilization After Surgery

**eTable 2**. Operational Definition of Opioid-Naivety in Studies Enrolling Only Opioid-Naive Individuals Before Surgery

**eTable 3.** Classification of Surgeries into Minor, Major, or Major and Minor Surgeries

**eTable 4.** Risk Factors for Prolonged Opioid Use After Surgery (Pooled Estimates After Leave-1-Out Analyses)

**eFigure 1.** Funnel Plot Assessing Publication Bias/Small-Study Effects

**eReferences.**

This supplementary material has been provided by the authors to give readers additional information about their work.

© 2020 Lawal OD et al. *JAMA Network Open.*

**eAppendix. Literature Search Strategy for MEDLINE**

1. Exposure

(("codeine"[MeSH Terms] OR "codeine"[All Fields]) OR ("fentanyl"[MeSH Terms] OR "fentanyl"[All Fields]) OR ("hydrocodone"[MeSH Terms] OR "hydrocodone"[All Fields]) OR ("hydromorphone"[MeSH Terms] OR "hydromorphone"[All Fields]) OR ("levorphanol"[MeSH Terms] OR "levorphanol"[All Fields]) OR ("meperidine"[MeSH Terms] OR "meperidine"[All Fields]) OR ("morphine"[MeSH Terms] OR "morphine"[All Fields]) OR ("oxycodone"[MeSH Terms] OR "oxycodone"[All Fields]) OR ("oxymorphone"[MeSH Terms] OR "oxymorphone"[All Fields]) OR ("pentazocine"[MeSH Terms] OR "pentazocine"[All Fields]) OR (("levopropoxyphene"[MeSH Terms] OR "levopropoxyphene"[All Fields] OR "propoxyphene"[All Fields] OR "dextropropoxyphene"[MeSH Terms] OR "dextropropoxyphene"[All Fields]) AND ("sufentanil"[MeSH Terms] OR "sufentanil"[All Fields])) OR ("tramadol"[MeSH Terms] OR "tramadol"[All Fields]) OR ("tapentadol"[MeSH Terms] OR "tapentadol"[All Fields]) OR ("buprenorphine"[MeSH Terms] OR "buprenorphine"[All Fields]) OR ("analgesics, opioid"[Pharmacological Action] OR "analgesics, opioid"[MeSH Terms] OR ("analgesics"[All Fields] AND "opioid"[All Fields]) OR "opioid analgesics"[All Fields] OR "opioid"[All Fields])) AND "Analgesics, Opioid"[Mesh]

2. Population of interest

((((("Surgical Procedures, Operative"[Mesh] OR (major[All Fields] AND ("surgical procedures, operative"[MeSH Terms] OR ("surgical"[All Fields] AND "procedures"[All Fields] AND "operative"[All Fields]) OR "operative surgical procedures"[All Fields] OR ("surgical"[All Fields] AND "procedures"[All Fields]) OR "surgical procedures"[All Fields]))) OR "Minor Surgical Procedures"[Mesh]) OR ("minor surgical procedures"[MeSH Terms] OR ("minor"[All Fields] AND "surgical"[All Fields] AND "procedures"[All Fields]) OR "minor surgical procedures"[All Fields])) OR ("General Surgery"[Mesh] AND "Surgical Procedures, Operative"[Mesh])) OR ("surgery"[Subheading] OR "surgery"[All Fields] OR "surgical procedures, operative"[MeSH Terms] OR ("surgical"[All Fields] AND "procedures"[All Fields] AND "operative"[All Fields]) OR "operative surgical procedures"[All Fields] OR "surgery"[All Fields] OR "general surgery"[MeSH Terms] OR ("general"[All Fields] AND "surgery"[All Fields]) OR "general surgery"[All Fields])) OR "Pain, Postoperative"[Mesh]

3. Outcomes

((((((((opioid prolonged use postoperative) OR opioid prolonged use surgery) OR opioid dependence postoperative) OR opioid persistent use postoperative) OR opioid chronic use postoperative) OR opioid dependence surgery) OR opioid chronic use postoperative) OR opioid dependence surgery) OR opioid persistent use surgery) OR opioid chronic use surgery

4. Limits

((((("humans"[MeSH Terms] OR "humans"[All Fields] OR "human"[All Fields])) NOT ("animals"[MeSH Terms:noexp] OR animal[All Fields]))) NOT ("child"[MeSH Terms] OR "child"[All Fields] OR "children"[All Fields])) OR ("animals"[MeSH Terms:noexp] OR animal[All Fields])

**Free text search for other databases**

© 2020 Lawal OD et al. *JAMA Network Open.*

opioid chronic use surgery, opioid persistent use surgery, opioid dependence surgery, opioid chronic use postoperative, opioid persistent use postoperative, opioid dependence postoperative, opioid prolonged use surgery, and opioid prolonged use postoperative

© 2020 Lawal OD et al. *JAMA Network Open.*

**eTable 1. Included Studies Categorized by Definitions for Assessing Opioid Utilization After Surgery**

| Operational definitions for opioid utilization after surgery | Author and Year |
|---|---|
| Two definitions: (a) using a trajectory model with 5 groups of subjects based on probability of filling an opioid during each of 12 consecutive 30-day period of follow-up; (b) filling an opioid in >=4, 6, or 8 months of follow-up | Bateman et al 2016[1] |
| Chronic opioid use: defined as ≥10 opioid fills, ≥90 consecutive days or ≥120 total days' supply in the one year following surgery after accounting for the first 90 postoperative days | Raebal et al,[2] 2014<br>Sun et al,[3] 2016<br>Politzer et al,[4] 2018<br>Schoenfeld et al,[5] 2017<br>Hansen et al,[6] 2017<br>Inacio et al,[7] 2016<br>Hadlandsmyth et al,[8] 2018 |
| Postoperative opioid assessed as the filling of opioid prescriptions within the first 90 days after surgery and one or more prescriptions of opioids 91 – 180 days after surgery | Clarke et al,[9] 2014<br>Brummett et al,[10] 2017<br>Johnson et al,[11] 2016<br>Rosenbloom et al,[12] 2017<br>Bennett et al,[13] 2019<br>Lindestrand et al,[14] 2015<br>Carroll et al,[15] 2012<br>Namba et al, [16] 2018<br>Goesling et al,[17] 2016<br>Rao et al, [18] 2018 |
| Prolonged opioid use assessed on the basis of the presence of ICD-9 diagnosis codes indicative for opioid dependence in medical records | Shah et al,[19] 2017 |
| Prolonged opioid use defined as filling ≥365 days of opioid prescriptions in the 24 months following surgery | Connolly et al,[20] 2017 |
| Prolonged use assessed by monthly trend of filling of opioids for up to 1 year after surgery | Bedard et al, [21] 2017<br>Kim et al,[22] 2017<br>Pugely et al,[23] 2018<br>Westermann et al,[24] 2017<br>Westermann et al,[25] 2019<br>Fuzier et al,[26] 2018 |
| Prolonged opioid use defined by filling of opioid prescriptions 3 months after surgery | Mulligan et al,[27] 2016<br>Rozet et al,[28] 2014 |
| Prolonged opioid use defined as ≥2 opioid prescriptions within 6 months of index surgery with ≥1 fill every 3 months and either total oral morphine equivalent or equal to 1150 or ≥ 39 days opioid supply | Swenson et al,[29] 2018 |
| Prolonged opioid use was assessed by questionnaire administered 2 and 5 years after index surgery | Singh and Lewallen,[30] 2012<br>Singh and Lewallen,[31] 2014<br>Singh and Lewallen,[32] 2010 |
| Postoperative opioid use was assessed by questionnaire administered about 1.3 years after index surgery | Valdes et al,[33] 2015 |

© 2020 Lawal OD et al. *JAMA Network Open.*

**eTable 2. Operational Definition of Opioid-Naivety in Studies Enrolling Only Opioid-Naïve Individuals Before Surgery**

| Definition of opioid-naivety | Author name |
|---|---|
| No fill of an opioid prescription in the 12 months preceding the index surgery | Sun et al,[3] 2016<br>Bateman et al 2016[1]<br>Clarke et al,[9] 2014 |
| No receipt of prescription opioid within the 6 months preceding the index surgery | Schoenfeld et al,[5] 2017 |
| No opioid prescriptions during the 11 months prior to surgery | Brummett et al,[10] 2017 |
| No opioid fills from 243 days to 31 days prior to index surgery | Swenson et al,[29] 2018 |
| No filling of an opioid prescription in the 11 months to 31 days prior to index surgery | Bennett et al,[13] 2019<br>Johnson et al,[11] 2016 |

© 2020 Lawal OD et al. *JAMA Network Open.*

**eTable 3. Classification of Surgeries into Minor, Major, or Major and Minor Surgeries**

| Source | Type of surgery | Surgery classification |
|---|---|---|
| Bateman et al 2016[1] | Cesarean delivery | Major |
| Raebal et al,[2] 2014 | Bariatric surgery | Major |
| Clarke et al,[9] 2014 | Coronary artery bypass graft surgery through sternotomy, open (thoracotomy) lung resection surgery, lung resection using video assisted thoracoscopic surgery, open colon resection surgery, minimally invasive (laparoscopic) colon resection surgery, open radical prostatectomy, minimally invasive (laparoscopic or robot assisted) radical prostatectomy, open total or radical hysterectomy, and minimally invasive (laparoscopic or robot assisted) total or radical hysterectomy. | Major |
| Shah et al,[19] 2017 | Urological surgery | Major |
| Sun et al,[3] 2016 | Total knee arthroplasty, total hip arthroplasty, laparoscopic cholecystectomy, open cholecystectomy, laparoscopic appendectomy, open appendectomy, cesarean delivery, functional endoscopic sinus surgery [fess], cataract surgery, transurethral prostate resection, and simple mastectomy | Both |
| Connolly et al,[20] 2017 | Lumbar spinal fusion surgery | Major |
| Bedard et al, [21] 2017 | Total knee arthroplasty | Major |
| Johnson et al,[11] 2016 | Hand surgery | Major |
| Schoenfeld et al,[5] 2017 | Spine surgery (discectomy, decompression, lumbar posterolateral arthrodesis, or lumbar interbody arthrodesis) | Major |
| Westermann et al,[24] 2017 | Rotator cuff repair | Major |
| Rosenbloom et al,[12] 2017 | Traumatic musculoskeletal injury and corrective surgery | Major |
| Brummett et al,[10] 2017 | Major surgical procedures included ventral incisional hernia repair, colectomy, reflux surgery, bariatric surgery, and hysterectomy.<br>Minor surgical procedures included varicose vein removal, laparoscopic cholecystectomy, laparoscopic appendectomy, hemorrhoidectomy, thyroidectomy, transurethral prostate surgery, parathyroidectomy, and carpal tunnel. | Major and minor |
| Fuzier et al,[26] 2018 | Carpal tunnel surgery | Minor |
| Carroll et al,[15] 2012 | Mastectomy, lumpectomy, thoracotomy, total knee replacement, or total hip replacement | Major |
| Pugely et al,[23] 2018 | Cervical spine surgery | Major |
| Politzer et al,[4] 2018 | Total knee arthroplasty | Major |
| Swenson et al,[29] 2018 | Hysterectomy | Major |
| Namba et al, [16] 2018 | Total knee arthroplasty | Major |
| Bennett et al,[13] 2019 | Post-bariatric body contouring surgical procedures | Minor |
| Westermann et al,[25] 2019 | Hip arthroscopy | Minor |
| Goesling et al,[17] 2016 | Total knee and total hip arthroplasty | Major |
| Lindestrand et al,[14] 2015 | Hip fracture surgery | Major |
| Mulligan et al,[27] 2016 | Ankle and foot reconstruction | Major |

© 2020 Lawal OD et al. *JAMA Network Open.*

| **Source** | **Type of surgery** | **Surgery classification** |
|---|---|---|
| Rao et al, [18] 2018 | Shoulder arthroplasty | Major |
| Singh and Lewallen,[32] 2010 | Primary total hip arthroplasty | Major |
| Singh and Lewallen,[30] 2012 | Primary total knee arthroplasty | Major |
| Singh and Lewallen,[31] 2014 | Revision total knee arthroplasty | Major |
| Valdes et al,[33] 2015 | Total joint replacement | Major |
| Rozet et al,[28] 2014 | Knee arthroscopy | Minor |
| Kim et al,[22] 2017 | Hip or knee arthroplasty | Major |
| Hansen et al,[6] 2017 | Total knee arthroplasty | Major |
| Inacio et al,[7] 2016 | Total hip arthroplasty | Major |
| Hadlandsmyth et al,[8] 2018 | Total knee arthroplasty | Major |

© 2020 Lawal OD et al. *JAMA Network Open.*

eTable 4. Pooled Risk Factors for Prolonged Opioid Use After Surgery (After Leave-1-Out Analyses) [a]

| Risk factors | Studies, n | References for all studies | Primary analyses Pooled OR (95% CI) | Removed study, reference | Pooled OR (95% CI), One-study removed |
|---|---|---|---|---|---|
| **Demographic characteristics** | | | | | |
| **BMI [b]** | | | | | |
| < 25 | NA | NA | 1 [Reference] | NA | 1 [Reference] |
| 25 – 29.9 | 4 | [8,30–32] | 1.04 (0.52, 2.10) | [30] | 1.26 (0.50, 3.14) |
| 30 – 34.9 | 3 | [30–32] | 1.21 (0.61, 2.38) | [31] | 1.46 (0.67, 3.20) |
| 35 – 39.9 | 3 | [30–32] | 0.63 (0.31, 1.30) | [31] | 0.78 (0.35, 1.79) |
| ≥ 40 | 3 | [30–32] | 0.98 (0.42, 2.33) | [32] | 0.80 (0.20, 3.51) |
| **Gender** | | | | | |
| Male | NA | NA | 1 [Reference] | NA | 1 [Reference] |
| Female | 14 | [4,6,8–11,16–18,20,21,26,32,33] | 1.16 (1.08, 1.25) | [4] | 1.15 (1.07, 1.24) |
| **Race/ethnicity** | | | | | |
| White | NA | NA | 1 [Reference] | NA | 1 [Reference] |
| African American | 7 | [2,8,10,16,18,19,29] | 1.02 (0.92, 1.13) | [18] | 1.06 (0.96, 1.17) |
| Asian | 4 | [10,16,18,19] | 0.68 (0.45, 1.03) | [19] | 0.82 (0.54, 1.25) |

© 2020 Lawal OD et al. *JAMA Network Open.*

| Risk factors | Studies, n | References for all studies | Primary analyses Pooled OR (95% CI) | Removed study, reference | Pooled OR (95% CI), One-study removed |
|---|---|---|---|---|---|
| Hispanic | 5 | 2,10,16,18,19 | 0.92(0.80, 1.05) | 19 | 1.02 (0.98, 1.06) |
| **Preoperative medication use** | | | | | |
| Antidepressants | 6 | 1–3,8,9,22 | 1.42 (1.11, 1.81) | 3 | 1.38 (1.05, 1.80) |
| Benzodiazepines | 5 | 1,3,8,9,22 | 1.53 (1.20, 1.95) | 1 | 1.32 (1.11, 1.59) |
| Opioids | 14 | 2,8,10,14,16–18,22–25,27–29 | 5.32 (2.94, 9.64) | 2 | 4.97 (2.70, 9.16) |
| **Substance use** | | | | | |
| Alcohol abuse | 8 | 3,10–12,19,21,22,27 | 1.55 (1.07, 2.25) | 22 | 1.70 (1.15, 2.52) |
| Tobacco | 10 | 1,2,10,11,13,19–22,27 | 1.55 (1.23, 1.96) | 20 | 1.61 (1.26, 2.05) |
| **Medical comorbidities** | | | | | |
| **Mental conditions** | | | | | |
| Anxiety | 9 | 2,10,12,13,16,18,30–32 | 1.14 (1.06, 1.23) | 12 | 1.17 (1.06, 1.29) |
| Depression | 15 | 2,3,7,12,16,18–21,23,25,29–32 | 1.54 (1.25, 1.91) | 29 | 2.62 (1.71, 4.02) |
| Mood disorders | 3 | 10,13,27 | 1.85 (1.11, 3.07) | 27 | 1.19 (1.07, 1.32) |
| Psychiatric disorders | 5 | 3,7,13,18,24 | 1.04 (0.95, 1.13) | 13 | 1.42 (0.72, 2.79) |
| Unspecified mental disorders | 5 | 10,11,13,16,26 | 1.45 (0.78, 2.68) | 11 | 1.10 (0.91, 1.33) |

© 2020 Lawal OD et al. *JAMA Network Open.*

| Risk factors | Studies, n | References for all studies | Primary analyses Pooled OR (95% CI) | Removed study, reference | Pooled OR (95% CI), One-study removed |
|---|---|---|---|---|---|
| **Pain conditions** | | | | | |
| Arthritis | 4 | 10,13,16,18 | 1.19 (0.93, 1.52) | 10 | 1.07 (0.90, 1.26) |
| Back pain | 11 | 1,6,7,10,16,18,21–24,33 | 2.05 (1.63, 2.58) | 6 | 1.81 (1.44, 2.28) |
| Chronic pain | 5 | 2,8,12,18,27 | 1.35 (1.04, 1.75) | 8 | 1.64 (1.04, 2.58) |
| Fibromyalgia | 7 | 1,16,18,21–24 | 1.43 (1.15, 1.79) | 21 | 1.36 (1.03, 1.78) |
| Migraine | 4 | 1,7,18,22 | 1.36 (1.02, 1.80) | 18 | 1.58 (0.92, 2.69) |
| Neck pain | 4 | 10,13,16,18 | 1.12 (1.02, 1.23) | 16 | 1.13 (0.97, 1.32) |
| Osteoarthritis | 3 | 16,18,26 | 1.03 (0.96, 1.09) | 16 | 0.99 (0.90, 1.10) |
| Unspecified pain disorders | 9 | 10,11,13,16–18,26,29,33 | 1.45 (1.21, 1.72) | 33 | 1.27 (1.16, 1.61) |
| **Other medical conditions** | | | | | |
| Diabetes | 5 | 7,9,18,26,27 | 1.09 (0.90, 1.31) | 9 | 1.15 (0.81, 1.63) |
| Liver disease | 4 | 7,16,18,19 | 1.23 (0.98, 1.54) | 18 | 1.47 (0.92, 2.34) |
| Pulmonary disease | 4 | 20,33 | 1.32 (1.07, 1.63) | 16 | 1.48 (0.93, 2.35) |
| Renal disease | 3 | 9,16,18 | 1.02 (0.97, 1.08) | 16 | 0.97 (0.89, 1.06) |
| Substance abuse | 11 | 1–3,8,11,12,16,18,21–23 | 1.58 (1.14, 2.21) | 21 | 1.37 (1.17, 1.60) |

© 2020 Lawal OD et al. *JAMA Network Open.*

[a] For each risk factor reported by three or more studies, we recalculated the pooled effect by omitting one study each time. When the conclusion of the recalculated pooled estimate did not differ (on the basis of statistical significance) from the initial analysis, we presented one of the recalculated estimates; otherwise, the recalculated estimate(s) yielding a conclusion different from the primary analysis was presented. In all instances, our analyses were performed using a random-effects model. [b] Calculated as weight in kilograms divided by height in meters squared.

© 2020 Lawal OD et al. *JAMA Network Open.*

**eFigure 1. Funnel Plot Assessing Publication Bias/Small-Study Effects**

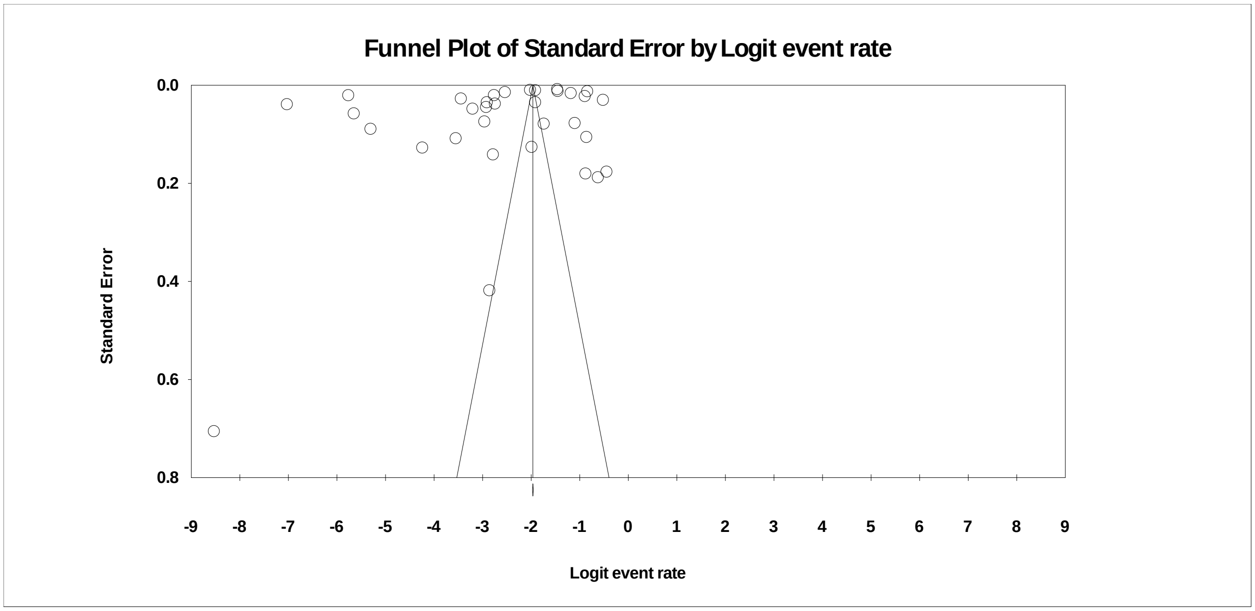


We plotted the standard error against the logit of the event rates of prolonged opioid use from the 33 eligible studies. Based on the results from the Egger's regression test, we have no evidence of small-study-effects (intercept: -20.99 [95%, -46.04% - 4.07%]; standard error: 12.28; *P* = .0976)

© 2020 Lawal OD et al. *JAMA Network Open.*

**eReferences.**


1. Bateman BT, Franklin JM, Bykov K, et al. Persistent opioid use following Cesarean delivery: patterns and predictors among opioid naïve women. *Am J Obstet Gynecol*. 2016;215(3):353.e1-353.e18. doi:10.1016/j.ajog.2016.03.016

2. Raebel MA, Newcomer SR, Bayliss EA, et al. Chronic opioid use emerging after bariatric surgery. *Pharmacoepidemiol Drug Saf*. 2014;23(12):1247-1257. doi:10.1002/pds.3625

3. Sun EC, Darnall BD, Baker LC, Mackey S. Incidence of and Risk Factors for Chronic Opioid Use Among Opioid-Naive Patients in the Postoperative Period. *JAMA Intern Med*. 2016;176(9):1286-1293. doi:10.1001/jamainternmed.2016.3298

4. Politzer CS, Kildow BJ, Goltz DE, Green CL, Bolognesi MP, Seyler TM. Trends in Opioid Utilization Before and After Total Knee Arthroplasty. *J Arthroplasty*. 2018;33(7S):S147-S153.e1. doi:10.1016/j.arth.2017.10.060

5. Schoenfeld AJ, Nwosu K, Jiang W, et al. Risk Factors for Prolonged Opioid Use Following Spine Surgery, and the Association with Surgical Intensity, Among Opioid-Naive Patients. *J Bone Joint Surg Am*. 2017;99(15):1247-1252. doi:10.2106/JBJS.16.01075

6. Hansen CA, Inacio MCS, Pratt NL, Roughead EE, Graves SE. Chronic Use of Opioids Before and After Total Knee Arthroplasty: A Retrospective Cohort Study. *J Arthroplasty*. 2017;32(3):811-817.e1. doi:10.1016/j.arth.2016.09.040

7. Inacio MCS, Hansen C, Pratt NL, Graves SE, Roughead EE. Risk factors for persistent and new chronic opioid use in patients undergoing total hip arthroplasty: a retrospective cohort study. *BMJ Open*. 2016;6(4):e010664. doi:10.1136/bmjopen-2015-010664

8. Hadlandsmyth K, Vander Weg MW, McCoy KD, Mosher HJ, Vaughan-Sarrazin MS, Lund BC. Risk for Prolonged Opioid Use Following Total Knee Arthroplasty in Veterans. *J Arthroplasty*. 2019;33(1):119-123. doi:10.1016/j.arth.2017.08.022

9. Clarke H, Soneji N, Ko DT, Yun L, Wijeysundera DN. Rates and risk factors for prolonged opioid use after major surgery: population based cohort study. *BMJ*. 2014;348:g1251. doi:10.1136/bmj.g1251

10. Brummett CM, Waljee JF, Goesling J, et al. New Persistent Opioid Use After Minor and Major Surgical Procedures in US Adults. *JAMA Surg*. 2017;152(6):e170504. doi:10.1001/jamasurg.2017.0504

11. Johnson SP, Chung KC, Zhong L, et al. Risk of Prolonged Opioid Use Among Opioid-Naïve Patients Following Common Hand Surgery Procedures. *J Hand Surg Am*. 2016;41(10):947-957.e3. doi:10.1016/j.jhsa.2016.07.113

12. Rosenbloom BN, McCartney CJL, Canzian S, Kreder HJ, Katz J. Predictors of Prescription Opioid Use 4 Months After Traumatic Musculoskeletal Injury and Corrective Surgery: A

Prospective Study. *The Journal of Pain*. 2017;18(8):956-963. doi:10.1016/j.jpain.2017.03.006

13. Bennett KG, Kelley BP, Vick AD, et al. Persistent Opioid Use and High-Risk Prescribing in Body Contouring Patients. *Plast Reconstr Surg*. 2019;143(1):87-96. doi:10.1097/PRS.0000000000005084

14. Lindestrand AG, Christiansen MLS, Jantzen C, van der Mark S, Andersen SE. Opioids in hip fracture patients: an analysis of mortality and post hospital opioid use. *Injury*. 2015;46(7):1341-1345. doi:10.1016/j.injury.2015.04.016

15. Carroll I, Barelka P, Wang CKM, et al. A pilot cohort study of the determinants of longitudinal opioid use after surgery. *Anesth Analg*. 2012;115(3):694-702. doi:10.1213/ANE.0b013e31825c049f

16. Namba RS, Singh A, Paxton EW, Inacio MCS. Patient Factors Associated With Prolonged Postoperative Opioid Use After Total Knee Arthroplasty. *The Journal of Arthroplasty*. 2018;33(8):2449-2454. doi:10.1016/j.arth.2018.03.068

17. Goesling J, Moser SE, Zaidi B, et al. Trends and predictors of opioid use after total knee and total hip arthroplasty. *Pain*. 2016;157(6):1259-1265. doi:10.1097/j.pain.0000000000000516

18. Rao AG, Chan PH, Prentice HA, et al. Risk factors for postoperative opioid use after elective shoulder arthroplasty. *J Shoulder Elbow Surg*. 2018;27(11):1960-1968. doi:10.1016/j.jse.2018.04.018

19. Shah AS, Blackwell RH, Kuo PC, Gupta GN. Rates and Risk Factors for Opioid Dependence and Overdose after Urological Surgery. *J Urol*. 2017;198(5):1130-1136. doi:10.1016/j.juro.2017.05.037

20. Connolly J, Javed Z, Raji MA, Chan W, Kuo Y-F, Baillargeon J. Predictors of Long-term Opioid Use Following Lumbar Fusion Surgery. *Spine*. 2017;42(18):1405-1411. doi:10.1097/BRS.0000000000002133

21. Bedard NA, Pugely AJ, Westermann RW, Duchman KR, Glass NA, Callaghan JJ. Opioid Use After Total Knee Arthroplasty: Trends and Risk Factors for Prolonged Use. *J Arthroplasty*. 2017;32(8):2390-2394. doi:10.1016/j.arth.2017.03.014

22. Kim SC, Choudhry N, Franklin JM, et al. Patterns and predictors of persistent opioid use following hip or knee arthroplasty. *Osteoarthr Cartil*. 2017;25(9):1399-1406. doi:10.1016/j.joca.2017.04.002

23. Pugely AJ, Bedard NA, Kalakoti P, et al. Opioid use following cervical spine surgery: trends and factors associated with long-term use. *Spine J*. 2018;18(11):1974-1981. doi:10.1016/j.spinee.2018.03.018

© 2020 Lawal OD et al. *JAMA Network Open.*

24. Westermann RW, Anthony CA, Bedard N, et al. Opioid Consumption After Rotator Cuff Repair. *Arthroscopy*. 2017;33(8):1467-1472. doi:10.1016/j.arthro.2017.03.016

25. Westermann RW, Mather RC, Bedard NA, et al. Prescription Opioid Use Before and After Hip Arthroscopy: A Caution to Prescribers. *Arthroscopy*. 2019;35(2):453-460. doi:10.1016/j.arthro.2018.08.056

26. Fuzier R, Serres I, Bourrel R, Palmaro A, Lapeyre-Mestre M. Analgesic Drug Prescription After Carpal Tunnel Surgery: A Pharmacoepidemiological Study Investigating Postoperative Pain. *Reg Anesth Pain Med*. 2018;43(1):19-24. doi:10.1097/AAP.0000000000000685

27. Mulligan RP, McCarthy KJ, Grear BJ, Richardson DR, Ishikawa SN, Murphy GA. Psychosocial Risk Factors for Postoperative Pain in Ankle and Hindfoot Reconstruction. *Foot Ankle Int*. 2016;37(10):1065-1070. doi:10.1177/1071100716655142

28. Rozet I, Nishio I, Robbertze R, Rotter D, Chansky H, Hernandez AV. Prolonged Opioid Use After Knee Arthroscopy in Military Veterans. *Anesthesia & Analgesia*. 2014;119(2):454. doi:10.1213/ANE.0000000000000292

29. Swenson CW, Kamdar NS, Seiler K, Morgan DM, Lin P, As-Sanie S. Definition development and prevalence of new persistent opioid use following hysterectomy. *Am J Obstet Gynecol*. 2018;219(5):486.e1-486.e7. doi:10.1016/j.ajog.2018.06.010

30. Singh JA, Lewallen DG. Predictors of use of pain medications for persistent knee pain after primary Total Knee Arthroplasty: a cohort study using an institutional joint registry. *Arthritis Res Ther*. 2012;14(6):R248. doi:10.1186/ar4091

31. Singh JA, Lewallen DG. Predictors of pain medication use for arthroplasty pain after revision total knee arthroplasty. *Rheumatology (Oxford)*. 2014;53(10):1752-1758. doi:10.1093/rheumatology/ket443

32. Singh JA, Lewallen D. Predictors of pain and use of pain medications following primary Total Hip Arthroplasty (THA): 5,707 THAs at 2-years and 3,289 THAs at 5-years. *BMC Musculoskelet Disord*. 2010;11:90. doi:10.1186/1471-2474-11-90

33. Valdes AM, Warner SC, Harvey HL, et al. Use of prescription analgesic medication and pain catastrophizing after total joint replacement surgery. *Semin Arthritis Rheum*. 2015;45(2):150-155. doi:10.1016/j.semarthrit.2015.05.004

© 2020 Lawal OD et al. *JAMA Network Open.*
